# Polyandrous Mexican Fruit Flies: Second Male Paternity and Biological Attributes of Transgenic Strains

**DOI:** 10.3390/insects13010005

**Published:** 2021-12-21

**Authors:** Betzabé Verónica-Murrieta, José Salvador Meza, Martha L. Baena, Gerardo Alvarado-Castillo, Diana Pérez-Staples

**Affiliations:** 1 Posgrado en Ciencias Agropecuarias, Facultad de Ciencias Agrícolas, Universidad Veracruzana, Xalapa 91050, Veracruz, Mexico; betzabe.veronicam@gmail.com (B.V.-M.); gealvarado@uv.mx (G.A.-C.); 2 Programa Operativo Moscas, SENASICA-SADER/IICA, Metapa de Dominguez 30860, Chiapas, Mexico; 3 Instituto de Investigaciones Biológicas, Universidad Veracruzana, Xalapa 91050, Veracruz, Mexico; mbaena@uv.mx; 4 Instituto de Biotecnología y Ecología Aplicada, Universidad Veracruzana, Xalapa 91050, Veracruz, Mexico

**Keywords:** *Anastrepha ludens*, Diptera, Tephritidae, Sterile Insect Technique

## Abstract

**Simple Summary:**

The Mexican fruit fly is an important pest of certain fruits. As part of its control, the Sterile Insect Technique (SIT) is used. This is an environmentally friendly means of control where insects are mass-reared, sterilized, and then released into areas where the pest is found. Sterile insects are dyed with a fluorescent pigment before release, to distinguish them from the wild population. The efficiency of this technique can be diminished if wild females first mate with a sterile male and then with a wild male. For the Mexican fruit fly, several transgenic strains have been developed that express a fluorescent protein marker for field detection, and are also used as reporters in the creation of strains with complex genetic systems. Here, we report on the biological attributes, mating competitiveness, and the proportion of paternity gained by the second male in twice-mated females with males from two transgenic strains. We found that the males expressing green florescence (443-G) had a better overall performance than the males expressing red fluorescence (419-R). We also found that females produced progeny mostly from the second male to mate with her. This could affect release ratios and diminish the efficiency of the SIT if wild females mate first with a sterile male but remate with a wild male, as she will then lay fertile eggs. These findings are helpful towards delimiting which strains can be used in the future, and determining the proportion of sterile to wild individuals that need to be released in affected areas, for the more efficient control of the Mexican fruit fly.

**Abstract:**

*Anastrepha ludens* (Diptera: Tephritidae), is a damaging agricultural pest. Currently, the Sterile Insect Technique (SIT) is used as part of its control. The SIT consists of the mass-rearing, sterilization, and release of insects in target areas. Sterile males mate with wild females, and prevent them from laying fertile eggs. However, even if females mate with sterile males, they can then remate with a second male. If this second male is wild, then this could reduce the efficiency of the SIT by producing viable offspring. The amount of progeny produced by second males (P2 values) for *A. ludens* is unknown. Here, we evaluated the biological attributes, mating competitiveness, and the proportion of male paternity gained by the second male, using strains that carry fluorescent marker genes and can be potentially used to develop transgenic sexing strains. Furthermore, the transgenic strains were irradiated, to test their ability to induce sterility in females. We found that the 443-G strain had significantly higher larval survival than the 419-R strain. No significant difference was found between the two strains in their mating probability with wild females. We found P2 values between 67 and 74% for the 419-R and the 443-G strain, respectively. Second male sperm precedence only decreased slightly after 12 days, suggesting that sperm from the first and second male is not mixing with time, but rather the second male’s sperm prevails. Furthermore, sterile 443-G males induced significantly higher sterility in females than sterile males from the 419-R strain. The apparent lower ability of the 443-G strain to inhibit female remating should be further investigated. Knowledge of the pre and postcopulatory performance of transgenic strains will help in understanding their potential for control.

## 1. Introduction

The troubling reduction in insect diversity worldwide has made it imperative to focus efforts on environmentally friendly means of pest control. In that sense, the area-wide management of tephritid flies through the Sterile Insect Technique (SIT) provides an opportunity to control pest insects such as the Mexican fruit fly, *Anastrepha ludens* [1,2]. The SIT is an autocidal control where insects are mass-reared, sterilized, usually through gamma radiation, and released in affected areas, where irradiated males will mate with wild females [3,4]. Sterile males transfer sperm with dominant lethal mutations, such that wild females will not be fertile [5]. However, if females are polyandrous, then release ratios will have to be adjusted to take into account multiple mating with fertile wild males after a sterile mating. In that sense, it is first important to determine the sperm precedence pattern for this species.

Before release, sterile pupae are placed in plastic bags and covered with a pigment powder [2]. When the adults emerge, the setae and head are pigmented with the powder. The fluorescent pigment allows the identification of sterile individuals from the wild [6,7]. This procedure has three disadvantages: first, the handling of the pupae, second, the use of hazardous pigments for human health, and third, the risk that some pupae are not adequately dyed and/or the pigment falls or wears off [8,9]. Once adults fall into the traps, they could remain up to a week in the liquid proteinaceous bait, and are then transported into alcohol for revision. Pigmentation of the head is revised using UV light, but sometimes the origin of the flies (wild or sterile) is not clear. Inadequate identification of wild or sterile populations can impact release ratios. Thus, in *A. ludens*, two transgenic lines were initially created to construct a female-specific embryonic lethal system that can be suppressed with tetracycline, which carries a gene that codes for a fluorescent protein; one of them produces a red fluorescent protein (DsRed) (419-R) and the other a green fluorescent protein (EGFP) (443-G) and the system is only activated when both strains are combined [10]. However, the advantage of using these strains separatedly is that fluorescence in the thorax can easily be identified though a fluorescent microscope, improving the reliability of field monitoring through phenotypic markers [11]. 

The use of transgenic strains in the context of SIT is promising, as males would be sterilized and unable to reproduce with local populations. However, the particular biological attributes of these strains should be studied, as this will affect the viability of such strains being mass-reared and sterilized. An essential step towards this goal is to evaluate their sexual competitiveness with wild females, since usually, mass-rearing and irradiation decrease the competitiveness of sterile males [12,13,14]. Consequently, the choice for producing a particular transgenic strain must be accompanied by tests of their biological attributes and sexual competitiveness after irradiation. 

Furthermore, SIT efficiency will be reduced if females are polyandrous. For the Mexican fruit fly, we know that females can mate with more than four males in laboratory conditions, and its refractory period can last between 24 h and 40 days, with a mean of 12 days after the first mating [15]. Polyandry can reduce the efficiency of SIT, in cases where females mate with a sterile and then a wild male. However, we do not know how paternity will be affected by multiple mating, and which of the two fathers will sire more progeny. In the context of SIT, it is important to understand the amount of fertility that is regained by a female that could mate with a sterile male but remates with a wild male. The use of transgenic strains provides an opportunity to visualize the proportion of paternity sired by the second male (P2) [16,17]. Moreover, the ability of transgenic strains in inducing sterility in polyandrous females has usually not been addressed. Studying the offspring production of transgenic males in sperm competition is crucial towards predicting their efficacy as population control agents, particularly in polyandrous species [18]. This in turn, should help in fine tuning release ratios, depending on the likelihood of females remating with wild males.

Here, we evaluated the biological attributes from larvae to adults for two transgenic *A. ludens* strains, and their sexual competitiveness with wild females. Furthermore, as a post-copulatory quality control test, we registered the amount of second male paternity (P2 values) gained in twice-mated females and the males’ ability to inhibit female remating. Lastly, we measured sterility induction in twice-mated females with either sterile transgenic males as the first mating, or second mating after a fertile mating. We expected one of the two transgenic strains to have better performance in terms of offspring production and sterility induction, and P2 values favoring the second male as reported for other tephritid species.

## 2. Materials and Methods

Studies were conducted at the Departamento de Genética, Laboratorio de Biología Molecular of the Programa Operativo Moscas (SENASICA-SADER/IICA) in Metapa de Dominguez, Chiapas, Mexico. Two transgenic lines were constructed with red (419-R) or green fluorescence (443-G) as follows: A germline transformation was performed by microinjection of the piggyBac transposase. The transgenic strain with red fluorescence carries the transgene pBXLII_[attP220_PUbDsRed.T3_tTA-Assrya] (419-R), which is inert and expresses the red fluorescent protein DsRed as visible marker. The transgenic gene with green fluorescence carries the transgene pBXLII_[PUbEGFP_TREhs43-CctraI-AlhidAla2_loxN-3xP3-FRT-AmCyan_lox2272_loxP_attP235], which is inert and expresses the green fluorescent protein EGFP as visible marker; both fluorescent markers are controlled by a constitutive polyubiquitin promoter. Only by combining both transgenes into the same individual can they produce a tetracycline-suppressible female-specific embryonic lethal system.

Flies were reared according to standard protocol [19], and adults were fed with hydrolyzed protein and sugar in a 1:3 proportion, and water. Fluorescence of both lines (443-G green fluorescence) (419-R red fluorescence) was corroborated the third day after emergence using a fluorescent microscope (LEICA MZFLII, YTP-green light and TXR-red light, Wetzlar, Germany). Flies were maintained in controlled conditions at 25 °C, 60% RH and a 12:12 light:dark cycle.

### 2.1. Biological Attributes 

Tests were carried out with wild type females and 443-G and 419-R males; as a control, males of the standard bisexual (meaning both males and females are produced and released) mass-reared strain of *A. ludens* were used. When adults reached sexual maturity (10 days of age), 100 adults from each strain were placed in 30 × 30 × 30 cm cages. Five panels (5 cm long PVC tube with a black linen cloth and a thin layer of silicon and water as an artificial ovipositing device) [20] were placed in each cage to ensure the oviposition of females and to carry out egg collection for 5 days; a subsample of three batches of 100 eggs were used for determine the proportion of eggs hatched. With another cohort, 100 neonate larvae were reared until adulthood. Larval survival, the number of pupae, and the number of male and female adults produced were scored for each transgenic and standard strain. Five replicates were carried out for each type of male.

### 2.2. Male Sexual Competition 

The sexual competitiveness of both transgenic strains was tested against wild females. Wild flies were collected from infested Matasano fruits, (*Casimiroa edulis*), in Metapa de Dominguez, Chiapas. Once wild females were 18 days of age and transgenic males were 8 days of age, they were placed in 30 × 30 × 30 cm cages. A few mango (*Mangifera indica*) leaves were placed inside each cage, thus providing flies with a more “natural” setting to perch. Ten wild females were placed with five 419-R males and five 443-G males in the same cage. Once matings formed they were carefully coaxed out with filter paper and placed inside a petri dish to finish the mating. The identity of the mated male was registered using a fluorescent microscope. Mating latency was registered as the time when males and females were placed together until they mated; copula duration and mating success were scored for each male. A total of 19 cages were observed for each type of male.

### 2.3. Paternity

For paternity analysis, virgin (unmated) males and females of 10 days of age were placed in individual cages in a 1:1 proportion (1 female with 1 male of either strain). Two sequences of crosses were carried between the two transgenic strains. Females from the standard strain of *A. ludens* were mated first to 443-G and then to 419-R males, while other females were mated first to 419-R and then to 443-G males. Copulations were observed between 3:00 pm and 7:00 pm, a mating was considered only if the copula duration lasted at least 15 min. Once copulations ended the first male was removed. Females that did not mate were discarded. Mated females were then provided a second male at 24 h. If she did not remate that day, then the male was removed and a new unmated male was provided at 48, 72, 96, or 120 h after the first mating. After the second mating, females were provided with a green agar sphere and a panel to produce offspring. Eggs were collected for 12 days during the peak oviposition period [21] and reared until adulthood. Once adults emerged from pupae they were checked under a fluorescent microscope (LEICA MZFLII, YTP-green light and TXR-red light, Wetzlar, Germany) and scored as offspring produced by either transgenic strain. This was repeated until we obtained 49 females who had mated first with the 443-G and then the 419-R male, and 42 females who had mated first with the 419-R and then the 443-G male (N = 91 females). Paternity values for the second male (P2 values) were calculated as the total number of progeny from the second male/total number of progeny from the first male × 100.

### 2.4. Induction of Sterility by Irradiated Males

Pupae from both transgenic strains were irradiated at 80 Gy with a Co60 Gammacell to obtain sterile males. Eye color and wing formation were checked prior to irradiation, to assure appropriate pupal development (two days before adult emergence) [22]. When males and females were 10 days of age, sterile or fertile males from both strains were mated either as first or second males to fertile females from the standard strain (four combinations). After the first mating, males were removed and mated females were provided with a different unmated male 24 h later. If they did not remate then, they were offered a second male at 48 h. Doubly mated females were placed with agar spheres and panels, described above. The number of eggs produced was registered for 95 females during 12 days and the proportion of eggs hatched per parental combination was observed 7 days after egg laying.

### 2.5. Statistical Analysis

Egg eclosion was analyzed by a nested ANOVA using the number of eggs hatched as dependent variables, and male type and repetition as independent variables; the cage was nested within male type and treated as a random variable, followed by the Tukey’s HSD as a post hoc test. The percentage of larval survival per type of male was analyzed by a GLM with a binomial distribution and a logit link, followed by contrasts as post hoc analysis. The number of pupae produced per male, and the total offspring produced, were analyzed by a GLM with a Poisson link and a log function, followed by contrasts as post hoc analysis. The proportion of male adults produced by each transgenic and bisexual strain was analyzed by a Binomial GLM with a Logit link. For all these analyses, the type of male and replicate were the independent variables.

The sexual competitiveness of males was analyzed by a paired t-test. Latency to mate was analyzed by ANOVA, and copula duration by a GLM with a normal distribution and link identity. The progeny produced by 419-R or 443-G males in the two parental combinations was analyzed by a GLM with binomial distribution and a logit link, using the total amount of progeny and the progeny produced by the 443-G male as dependent variables; the sexual refractory period (time elapsed between one mating and the next) and the interaction with the parental combination was also included in the model as independent variables, adding an overdispersion parameter to the model. The sexual refractory period was further analyzed by contingency tables according to the male combination. Total amount of progeny produced over time was analyzed by a repeated measures ANOVA.

For sterile males, we analyzed the number of offspring (fecundity and fertility) for each paternal combination per day by a repeated measures ANOVA, followed by post hoc contrast tests. Total fertility for irradiated males was analyzed by a GLM with a binomial distribution and a logit link, followed by contrasts as post hoc tests. The probability of remating was analyzed by a likelihood ratio χ^2^ contingency table. Statistical analysis was carried out in JMP v.9.0.0 (SAS institute, Inc. 2010, Cary, NC, USA).

## 3. Results

### 3.1. Biological Attributes

Egg eclosion was significantly higher for the two transgenic strains than for the bisexual strain (F = 14.73, df = 2, 6, *p* = 0.0048 male type, F = 2.66, df = 4, 32, *p* = 0.0505). Post hoc tests revealed no significant differences between the two transgenic strains (Table 1). The proportion of larvae that survived was also significantly higher for the standard strain than for either of the two transgenic strains, while the 443-G strain had higher survival than the 419-R strain (χ^2^ = 105.14, df = 2, 68, *p* < 0.0001, replicate χ^2^ = 11.01, df = 4, 68, *p* = 0.027) (Table 1). Almost all larvae transformed into pupae, thus the same pattern emerged for total pupae produced. The standard strain produced more pupae than either transgenic strain (χ^2^ = 36.93, df = 2, 68, *p* < 0.0001, replicate χ^2^ = 3.92, df = 4, 68, *p* = 0.416). Post hoc tests found significant differences between all strains (standard strain produced an average of 69.5%, 443-G produced 62.7%, while the 419-R strain produced only 55.9% of pupae). We found no significant differences in the proportion of males produced either by females mated to transgenic or standard males (type of male χ^2^ = 2.95, df = 2, 68, *p* = 0.229, replicate χ^2^ = 2.90, df = 4, 68, *p* = 0.575) (Table 1). The standard strain produced overall significantly more offspring than the 443-G or 419-R strain (χ^2^ = 25.62, df = 2, 68, *p* < 0.0001, replicate χ^2^ = 4.04, df = 4, 68, *p* = 0.400). Post hoc tests revealed that the 443-G strain produced more offspring than the 419-R strain (Table 1).

### 3.2. Male Sexual Competitiveness 

When competing against each other for wild females, there was no significant difference in the amount of copulations obtained by males from either transgenic strain, with both strains securing on average 40% of the copulations per cage (t-Ratio = −0.515, df = 18, *p* = 0.306). There was no significant difference between males in how fast they procured matings (latency to mate, F = 0.0004, df = 1, 60, *p* = 0.984, replicate F = 1.064, df = 18, 60, *p* = 0.409). 419-R males copulated for an average of 61.43 ± 4.22 min in comparison to longer matings by the 443-G males (71.42 ± 3.95), although this fell short of significance (copula duration, χ^2^ = 3.50, df = 1, 60, *p* = 0.061, replicate χ^2^ = 32.86, df = 18, 60, *p* = 0.017).

### 3.3. Second Male Paternity

**Figure 1:**
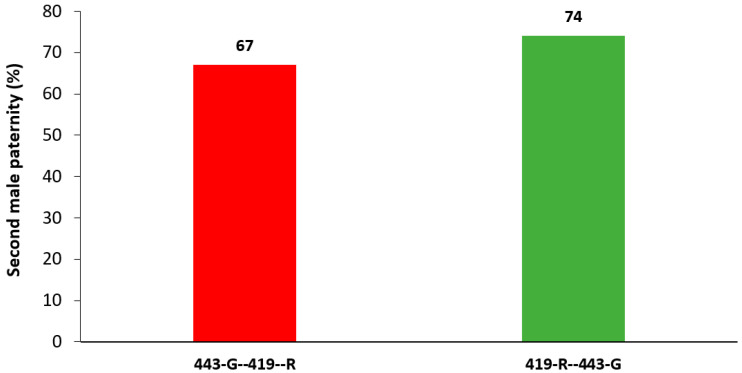
Second male paternity. Percentage of *A. ludens* red fluorescent offspring produced by a female mating first with a 443-G male and then mating with a 419-R male (red bar), or green fluorescent offspring produced by females mating first with a 419-R male and then a 443-G (green bar) male.

**Figure 2:**
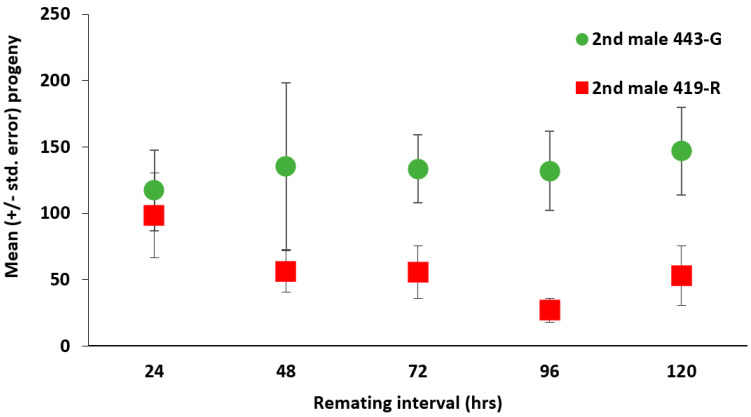
Mean (±std. error) progeny produced by *A. ludens* females mating first with a 443-G male and then mating with a 419-R male (red squares), or females mating first with a 419-R male, and then a 443-G (green circles) according to the female sexual refractory period. Females were offered the second male 24, 48, 72, 96, or 120 h after first mating. There were only significant differences between strains. GLM Binomial distribution χ^2^ = 33.074, df = 1, 81, *p* < 0.001.

**Figure 3:**
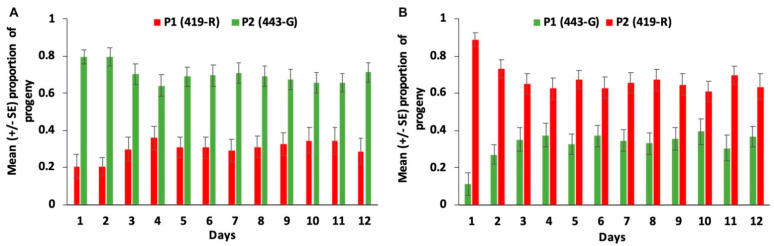
Mean (±std. error) green or red fluorescent progeny produced over a 12-day period by *A. ludens* females mating either first with (**A**) 419-R male (first male P1 red bar), and a 443-G male (second male P2 green bar); or (**B**) a 443-G male (first male P1 green bar) and a 419-R (second male P2 red bar). Each bar represents progeny attributed to each male.

For both strains there was a high second male paternity (Figure 1). Males from the 443-G strain were more likely to produce offspring when they were the second male to mate than when 419-R males were the second to mate (χ^2^ = 33.074, df = 1, 81, *p* < 0.001, Figure 2); the female sexual refractory period did not have a significant effect on the total amount of offspring they produced (χ^2^ = 0.294, df = 4, 81, *p* = 0.990), nor the interaction between parental combination and the remating interval (χ^2^ = 2.417, df = 4, 81, *p* = 0.660). Female sexual refractory period did not depend on male mating combination (χ^2^ = 2.279, df = 4, 91, *p* = 0.685). There was no significant effect of the parental combination on daily offspring production (F = 0.629, df = 11, 979, *p* = 0.805), however offspring production that was attributed to the second male varied significantly across 12 days for both strains (F = 2.541, df = 11, 979, *p* = 0.004). That is, males from the 443-G strain produced 89% of offspring as the second males on day 1 after mating, and this decreased by 15% on day 12; in contrast, males from the 419-R strain lost 28% of paternity by day 12 (Figure 3).

### 3.4. Induction of Sterility by Irradiated Males

**Figure 4:**
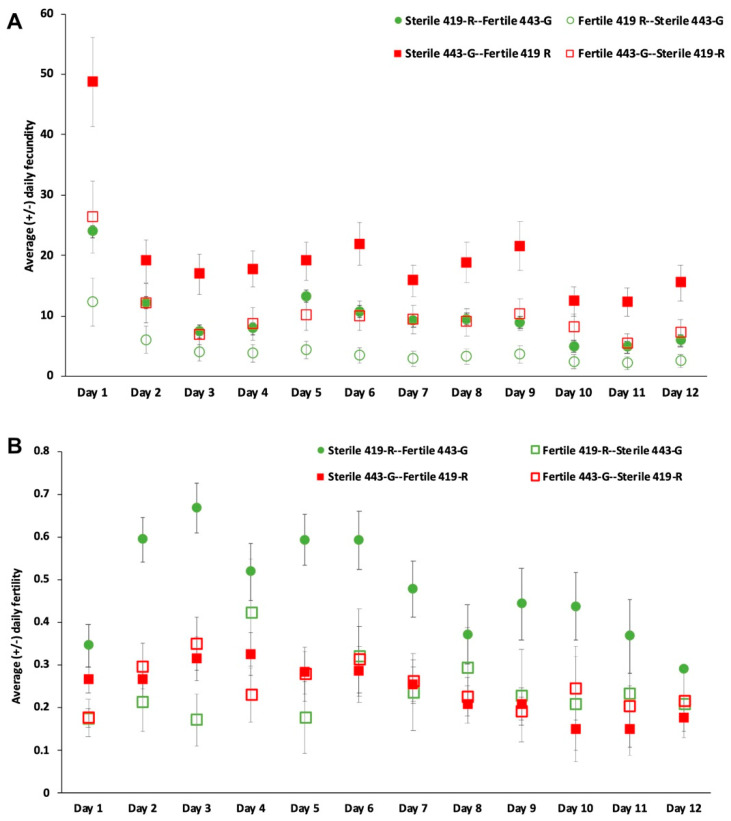
Daily average (± std. error) of (**A**) fecundity (eggs laid), and (**B**) fertility (eggs hatched/eggs laid) for *A. ludens* females that mated first with either a sterile 419-R male and then a fertile 443-G male (green circle); a fertile 419-R and then a sterile 443-G male (open green circle); a sterile 443-G male and then a fertile 419-R male (red circle); or a fertile 443-G and then a sterile 419-R male (open red circle).

There was a statistically significant difference in daily fecundity based on parental combination (F = 1.495, df = 33, 902.24, Wilks Lambda = 0.8548, *p* = 0.0371). Post hoc tests revealed significant differences in the daily fecundity between 419-R and 443-G sterile males when they were the first male to mate, and no significant difference when they were the second male to mate. Daily fecundity was higher for females that mated first to 443-G sterile males and then remated with 419-R fertile males when compared to any of the other combinations (Figure 4). In all cases, daily fecundity and fertility were highest on the first day of oviposition and decreased with time (Figure 4). 

**Figure 5:**
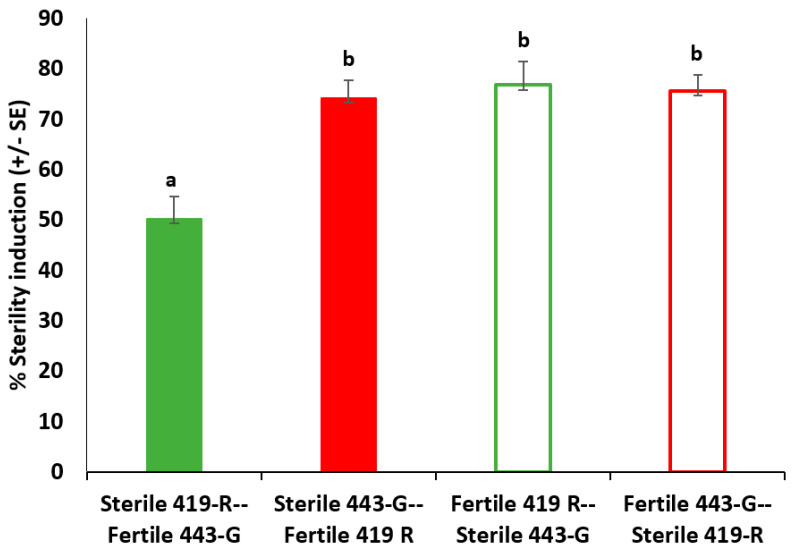
Sterility induction (percentage of fertility decreased: 1/(total fertile eggs/total eggs laid) × 100 (±std. error) for twice mated *A. ludens* females. Matings first with either a sterile 419-R male and then a fertile 443-G male (green bar); a fertile 419-R and then a sterile 443-G male (open green bar); a sterile 443-G male and then a fertile 419-R male (red bar); or a fertile 443-G and then a sterile 419-R male (open red bar). Different letters indicate significant differences. Sterile 443-G males were significantly more likely to induce sterility when mating first when compared to sterile 419-R males.

Total fertility was significantly different according to male mating type (χ^2^ = 26.020, df = 3, 90, *p* < 0.001). Importantly, 443-G sterile males were able to induce sterility in females when first mating with them, even after a fertile mating (25% of eggs being fertile), in contrast to 419-R sterile males, with close to 50% of eggs being fertile after females remated with a fertile male (Figure 5). 

**Figure 6:**
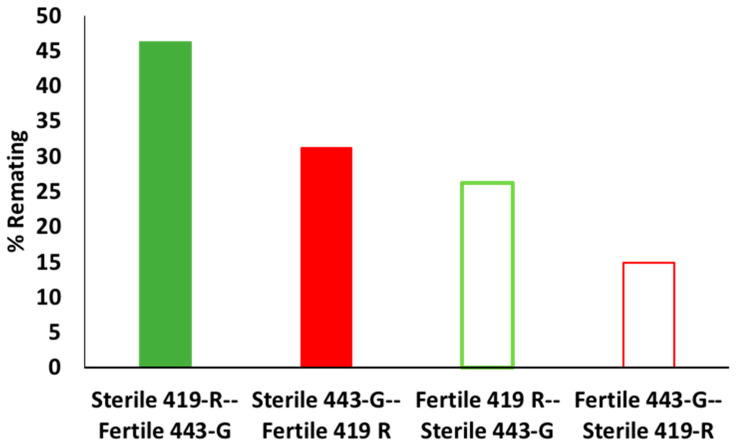
Percentage of *A. ludens* females remating. Matings where first with either a sterile 419-R male and then a fertile 443-G male (green bar); a fertile 419-R and then a sterile 443-G male (open green bar); a sterile 443-G male and then a fertile 419-R male (red bar); or a fertile 443-G and then a sterile 419-R male (open red bar).

Female refractory period also had a significant effect on fertility (χ^2^ = 5.637, df = 1, 90, *p* = 0.0176). The fertility of females that remated 24 h after mating was 60%, versus 40% when remating 48 h after the initial copulation (regardless of male type). There was also a statistically significant difference in the likelihood to remate by females depending on the male type (χ^2^ = 19.678, df = 3, *p* = 0.0002). Importantly, 443-G sterile males were less able to inhibit female remating when compared to other males, as the highest number of rematings were for females that mated with a sterile 443-G male and then were offered a fertile 419-R male (Figure 6). There was no significant effect of the mating combination on the female refractory period (24 or 48 h to remating) (χ^2^ = 5.388, df = 3, *p* = 0.1455).

## 4. Discussion

The Sterile Insect Technique is an environmentally friendly means of control. However, certain procedures, such as the marking of sterile males with a fluorescent pigment, can have negative consequences on the quality of pupae, an increased risk to human health from the use of pigments, and the possibility that some sterile adults will not be dyed properly [8,9]. Thus, the use of fluorescent transgenic strains is an alternative to the use of these pigments. Testing the pre and postcopulatory performance of these strains is necessary to understand their efficiency in future control methods. 

Ideally, these strains will need to be produced within a mass-rearing context, thus it is important to measure their biological attributes. Here, we show that the 443-G strain has better overall quality in terms of larval survival and pupal production. However, we cannot rule out a possible toxic effect of the tTA in this strain. If the tTA is transferred in the ejaculate, then higher female offspring mortality would have been observed, yet there was no discernable differences in the sex ratio of the progeny produced. Except for egg eclosion, the biological attributes of the standard bisexual strain were better when compared to both transgenic strains, as has been reported for other transgenic strains of *A. ludens* 11M3, 1M1 and 3F6 [19,23]. However, this is to be expected, as this strain has been under production for approximately 168 generations [24], so it is well-adapted to mass-rearing. We found no difference in the ability of males from either strain to gain copulations with wild females, their latency to mate, or copula duration. However, further studies should test them in competition against wild males as well. Other studies in *C. capitata* or *Bactrocera oleae* have found a reduced competitiveness against wild males, but no strong wild female discrimination against Genetic Sexing Strains or Transgenic Sexing Strains [25,26].

The efficiency of SIT depends on a sterile male mating with a wild female, nevertheless, if females then remate with a wild fertile male, then their fertility could be restored. Here, second male paternity was found to be between 67 and 74%, which is comparable to other tephritid species studied. In *Rhagoletis pomonella*, P2 values range from 79 to 93% [27], for *Drosophila melanogaster* 80% [16], and for *Bactrocera tryoni* 72% (calculated from [28]). In *Ceratitis capitata*, P2 values range between, 69, 67, or 75%, depending on transgenic strains [17,29]. All of these studies point towards a strong second male paternity advantage when there is a double mating. Further proof of strong second male sperm precedence for *A. ludens* was evident when females mated first to a sterile and then to a fertile male. In this case, female fertility was regained and was particularly high when females remated with a fertile 443-G male. Although the opposite mating (females mated first with a sterile 443-G, and then a fertile 419-R) did not show such high fertility, this may be confounded by an overall lower performance of 443-G males, including, possibly, lower sperm transfer. Indeed, P1 values (number of progeny of the first male/P2 values) were 33% for 443-G males and only 26% for 419-R males. Additional indications of a more competitive ejaculate by the 443-G male were also evident in inducing female sterility. Sterile males from the 443-G strain were more likely to induce sterility when mating first when compared to sterile males from the 419-R strain.

Sperm precedence patterns in *C. capitata* have found that although sperm from two males are stratified in the spermathecae immediately after mating, by seven days of age, they are mixed, and sperm from the first male is increasingly used over time [17,18]. While we do not have data on the pattern of sperm use for a polyandrous *A. ludens* female, we can make some inferences based on the progeny produced, although we cannot rule out higher progeny mortality in one of the strains. Here, sperm stratification and not mixing was more evident, as there was strong second male paternity the day after first mating for both strains (87%), and by day seven this paternity only slightly decreased (63 and 73% for the 419-R and 443-G strains, respectively). Comparatively, in *C. capitata* females, second male paternity decreased from approximately 70% on day one to 60% on day seven for females remating with tGFP1 strain males, and from 82 to 59%, respectively, for females remating with DsRedEx1 strain males [17]. Other studies, [29,30] also report a decrease in second male paternity over time, with the first male becoming more dominant. For example, P2 values for wild *C. capita* males decreased from 53.2 to 29% from day 1 to day 10 after mating, and for Vienna 8 1260 males, from 33.33 to 2.1% [30]. In contrast, in *A. ludens,* this dramatic decrease in second male paternity was not seen; P2 values on day 1 were 87.9 vs. 72.0% on day 10 for 443-G males as the second male, and 87.7% on day 1 vs. 66% on day 10 for 419-R males as second males. For both species, it is clear that even though second male paternity prevails, the paternity of the first male increases with time. However, the increase of P1 values seems to be more evident in *C. capitata* than in *A. ludens*. In the case of *A. ludens,* we have information up to 12 days, during which P2 values remain constant, although slightly decreased from day one. 

Differences in second male paternity between species may be due to a higher sperm storage capacity in *A. ludens* when compared to *C. capitata. Anastrepha ludens* has three spermathecae, compared to only two for *C. capitata*, and stores more sperm in the ventral receptacle when compared to *C. capitata* (26 vs. 15%), respectively, [31,32,33,34]. Sperm is first deposited in the ventral receptacle, where they are used for fertilization, and this storage site is continuously replenished from the spermathecae [34,35]. Given the increased sperm storage in the ventral receptacle, or fertilization chamber, for *A. ludens,* in addition to another spermathecae, it is likely that the sperm from the second male will be used for fertilization for a longer time in *A. ludens* when compared to *C. capitata,* where sperm have been found to mix after seven days [17]. Indeed in *A. suspensa,* only the ventral receptacle has been found to have mixed sperm from two males; the spermathecae did not store mixed sperm, and the spermathecae of doubly mated females stored mostly sperm from the second male [36]. Thus, these results suggest that sperm mixing in *Anastrepha* may be relatively lower when compared to *C. capitata,* as female sperm storage organs provide an opportunity for discrete sperm storage. Females could then bias paternity towards the second male. 

We found that polyandrous females that mated first to a sterile male and then a fertile male would indeed regain fertility. For example, when females mated first to the 419-R sterile male and then the 443-G fertile male, roughly 50% of eggs laid were fertile; this only decreased to 25% when females were mated first to a sterile 443-G male and then remated with a 419-R fertile male. Female fertility remained high with a second fertile mating (up to 67%), decreasing to 28% by day 12 for the 443-G strain, and from 26% for the first day down to 17% for day 12 for the fertile 419-R strain. Consistent with better overall performance, the 443-G strain induced a higher percentage of female sterility when compared to the 419-R male. When females mated first with a sterile 443-G male, they had similar fertility to when females mated first with a fertile male. The only negative aspect of the 443-G strain was their lower ability to inhibit female remating. When females mated with 443-G sterile males they were more likely to remate (46%) than females that had mated first with a 419-R sterile male (31%). However, these results should be verified with wild females, as females from the standard bisexual strain have been shown to remate more often than wild females [37]. This type of sterility induction test, where females mate first with a sterile male and then a second fertile male, is recommended as a post-copulatory evaluation that can indicate the ejaculate competitiveness of sterile males.

Despite *A. ludens* being an economically important pest species, this is the first report of second male paternity values for this polyandrous species. P2 values biased towards the second male are not surprising, yet there do seem to be differences when compared to other tephritid pest species, in terms of sperm mixing within sperm storage organs [38]. While we do not have data on sperm storage patterns for multiply mated *A. ludens*, the first male does not seem to gain much paternity from the first to the twelfth day of oviposition, which indicates low sperm mixing and dominance of the second male’s ejaculate. While it could be argued that females used up the sperm of the first male during their refractory period (between 24 to 120 h), P2 values for the 443-G strain did not vary according to the time that had elapsed between the first and the second mating. In the context of SIT, this implies that release ratios should take into account the fact that wild *A. ludens* females are polyandrous and their fertility could be restored if they find and mate with a wild male. Polyandry in wild *A. ludens* females may be more worrisome than for *C. capitata,* given this high second male sperm precedence and the apparent lack of sperm mixing throughout time. Finally, further research could be carried out on the use of the 443-G strain, as it had better overall performance in terms of rearing, as well as sexual and ejaculate competitiveness. 

## Figures and Tables

**Table 1:** Mean (±) s.e. of biological attributes of three strains of *Anastrepha ludens.* Different letters represent significant differences (post hoc contrast tests, and Tukey’s HSD for egg eclosion). No letters indicate that there was no significant difference in that attribute according to male type. Egg eclosion: number of neonate larvae/total eggs incubated (%); Larval survival: number of 3rd instar larvae; Pupae produced: number of larvae transformed to pupae; Males, Females produced: number of adults of each sex that emerged from the pupae; Total offspring: sum of males and females produced.

Male Type	Egg Eclosion (%)	Larval Survival	Pupae Produced	Males Produced	Females Produced	Total Offspring
Standard strain	82.0 a	70.4 ± 3.02 a	69.5 ± 2.89 a	32.1 ± 1.60	32.6 ± 1.49	64.7 ± 2.70 a
419-R	86.8 b	56.5 ± 3.61 b	55.9 ± 3.60 b	28.2 ± 1.85	25.6 ± 1.96	53.7 ± 3.66 b
443-G	89.4 b	63.3 ± 4.46 c	62.7 ± 4.48 c	31.1 ± 2.38	28.5 ± 2.29	59.6 ± 4.28 c

## Data Availability

Data will be available from the corresponding author upon reasonable request.
